# Pattern of utilization, disease presentation, and medication prescribing and dispensing at 51 primary healthcare centers during the Hajj mass gathering

**DOI:** 10.1186/s12913-022-07507-3

**Published:** 2022-02-03

**Authors:** Saber Yezli, Yara Yassin, Abdulaziz Mushi, Yasir Almuzaini, Anas Khan

**Affiliations:** 1 The Global Centre for Mass Gatherings Medicine, Ministry of Health, Riyadh, Saudi Arabia; 2 Department of Emergency Medicine, College of Medicine, King Saud University, Riyadh, Saudi Arabia

**Keywords:** Primary care, WHO drug use indicators, Diagnosis, Health service utilization, Prescribing pattern, Disease

## Abstract

**Background:**

The majority of pilgrims seeking healthcare during Hajj are seen at primary healthcare centers (PHCCs). Data on the utilization of these facilities during Hajj can aid in directing optimal health services delivery and allocation of resources during the pilgrimage.

**Method:**

We investigated the pattern of disease presentation, caseload, and medication prescribing and dispensing at 51 PHCCs during the 2019 Hajj. Data on patients’ demographics, diagnoses, and prescribed medications were retrieved from each PHCC’s electronic records and analyzed. Data were also used to calculate six of the World Health Organization (WHO) indicators for drug use at these facilities.

**Results:**

Data were captured for 99,367 patients who were mostly Hajj pilgrims (95.4%), male (69.1%) from the Eastern Mediterranean (60.8%) and had a mean age of 46.6 years (SD = 14.9). Most patients (85.2%) were seen in Mina and towards the end of Hajj. The majority of patients (96.0%) had a single diagnosis; most commonly, respiratory (45.0%), musculoskeletal (17.2%), and skin (10.5%) diseases. Patients were prescribed 223,964 medications, mostly analgesics (25.1%), antibacterials for systemic use (16.5%), anti-inflammatory and antirheumatic products (16.4%), and cough and cold preparations (11.9%). On average, 2.25 (SD = 0.94) medications were prescribed per consultation, with low (1.3%) prevalence of polypharmacy. An antibiotic and an injectable were prescribed in 43.6 and 2.67% of patient encounters, respectively. Most (92.7%) of the prescribed drugs were actually dispensed, in an average time of 8.06 min (SD = 41.4). All PHCCs had a copy of the essential drugs list available, on which all the prescribed drugs appeared.

**Conclusion:**

Respiratory illnesses are the main reason for PHCCs visits during Hajj, and analgesics and antibiotics are the most common medications prescribed to pilgrims. Our results, including the calculated WHO drug use indicators, contribute to evidence-based optimization of primary healthcare services during Hajj.

## Introduction

The Hajj pilgrimage to Makkah, Kingdom of Saudi Arabia (KSA), is one of the five pillars of Islam and is a religious duty for every Muslim who is physically and financially able to perform it [1]. Hajj is undertaken over a few specific days during the 12th month (DulHija) of the Islamic calendar, although most pilgrims spend longer times in Makkah and/or visiting the holy city of Medina. During the Hajj ritual days, pilgrims move to the holy site of Mina where they spend a few days, including 1 day (9th DulHija) in the desert plain of Arafat and a short stay at Muzdalifah area [1]. Each year, 2–3 million pilgrims from around the world perform the physically demanding Hajj rituals in crowded conditions, often outdoors [1, 2]. As such, the mass gathering presents a number of public health risks, including the transmission of infectious diseases, exacerbation of underlying health conditions, accidents and injuries, as well as environmental-related health conditions [3].

Saudi authorities use a well-coordinated and inter-sectoral approach to orchestrate the public health planning and management of Hajj, which includes addressing the increased demand for health services during the event. Free healthcare is provided for Hajj pilgrims through numerous permanent and seasonal (only operational during the Hajj season) hospitals and primary healthcare centers (PHCCs) in Makkah and Medina. In 2018, 21 permanent and eight seasonal hospitals were available, in addition to 33 permanent and 106 seasonal PHCCs [2]. Of the latter, 93 were located in the holy sites of Mina, Arafat and Muzdalifah. Pilgrims of certain countries may also access healthcare through their own Hajj medical missions [4].

Data on the pattern of diseases presentation and medication use at healthcare facilities during Hajj can assist public health planning for the event and direct optimal resources allocation and services delivery for pilgrims. Such data can also highlight gaps in the health system that need addressing for the provision of better healthcare in general. Several studies reported on the pattern of diseases and medication use among pilgrims attending hospitals during Hajj [5–8]. However, few reported on pilgrims attending PHCCs [9, 10], which represent the majority of those seeing healthcare during the event. For instance, Saudi Ministry of Health statistics indicates that during the 2018 Hajj season, 586,587 pilgrims visited PHCCs compared to 53,038 and 98,163 who visited hospitals’ ERs and outpatient departments (OPDs), respectively [2]. The above studies were conducted over a decade ago and were focused on PHCCs located in Mina. In addition, medication use and prescribing patters in PHCCs during Hajj were not previously investigated using the measurable World Health Organization (WHO) drug use indicators for health facilities [11].

This study aims to determine the pattern of disease presentation, caseload, and medication prescribing and dispensing at seasonal PHCCs in Mina, Arafat, and Muzdalifah, during Hajj. This is to provide an evidence base to direct optimal health services delivery and allocation of resources during the pilgrimage.

## Methods

### Study design and setting

We conducted a retrospective, descriptive cross-sectional study during the 2019 Hajj. The study period was from the 7th–13th DulHija 1440 H corresponding to 8th–14th August 2019. Data were obtained from 51 PHCCs located in the holy Hajj sites of Mina, Arafat and Muzdalifah, in Makkah, KSA. These represent 55% of PHCCs in these holy sites.

### Data collection and management

Data were retrieved from the PHCCs’ electronic health records and included demographic information of the patients (age, gender, nationality, residency, and Hajj status), diagnoses, and prescribed medications (name, dosage, and dates and times of prescribing and dispensing). Patients’ diagnoses were grouped and reported as chapters of the 10th revision of the International Classification of Diseases (ICD-10). We assigned the medications’ therapeutic classes based on the Anatomical Therapeutic Chemical (ATC) classification system of the WHO Collaborating Center on Drug Statistics Methodology (https://www.whocc.no/). Polypharmacy and excessive polypharmacy were defined as prescribing ≥5 medications and ≥ 10 medications per encounter, respectively [12].

### Data analysis

Descriptive statistics (e.g., mean, standard deviation (SD), frequencies, percentages) were computed for variables as appropriate. Medications prescribing patterns at the PHCCs were evaluated using the WHO core drug use indicators for health facilities [11]. Based on the available data, six indicators were calculated: four *prescribing indicators* (average number of drugs per encounter, percentage of encounters with an antibiotic or an injection prescribed and percentage of drugs prescribed from essential drugs list or formulary); one *patient care indicator* (percentage of drugs actually dispensed) and one *health facility indicator* (availability of a copy of essential drugs list or formulary). The average time from medication prescribing to dispensing was also calculated as a proxy to a second WHO patient care indicator; average dispensing time, as reported previously [5]. Data were analyzed using SPSS 22.0 (SPSS Inc., Chicago, USA) statistical package.

## Results

### Patients’ characteristics

**Fig. 1:**
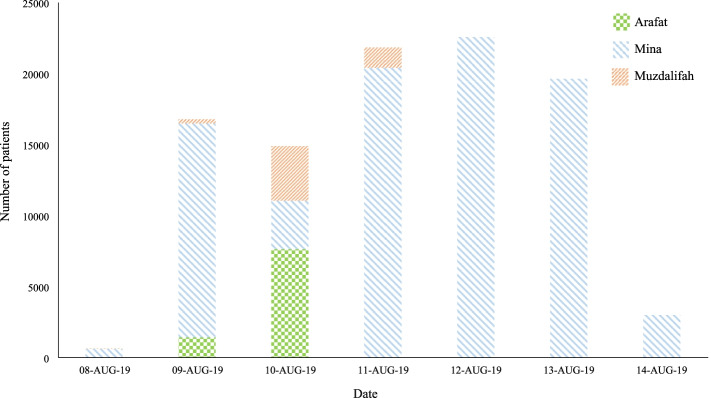
Number of patients attending 51 seasonal primary healthcare centers in the holy sites during the 2019 Hajj

Data were captured for 99,367 patients who attended 51 PHCCs in the holy sites of Mina, Arafat and Muzdalifah during the study period (Table 1). Patients originated from 186 countries and territories worldwide, mainly from the Eastern Mediterranean (60.8%), Africa (21.3%) and South-East Asia (10.9%) regions. The most represented countries were Egypt (18.8%), Nigeria (10.9%), Pakistan (9.1%), Saudi Arabia (8.1%) and India (6.3%). Most patients were Hajj pilgrims (95.4%), non-KSA residents/nationals (87.7%) and male (69.1%). Age was reported for 73.3% of patients with a mean age of 46.6 years (SD = 14.9, range 0–106 years). Most patients (85.2%) were seen at the 25 PHCCs located in Mina (Table 1). Pilgrims attended the PHCCs from the 8th–14th August (7th–13th DulHija) with the highest attendance from 11th–13th August (10th–12th DulHijja) (Fig. 1). The largest caseload at Arafat PHCCs was on the 10th August (9th DulHija).

**Table 1:** Demographic characteristics of patients attending the 51 seasonal primary healthcare centers in the holy sites during the 2019 Hajj

Variable	n	%
**Gender**	**99,293**	
Male	68,587	69.1
Female	30,706	30.9
**Age (Years)**	**72,913**	
≤ 19	2249	3.1
20–34	13,079	17.9
35–49	23,800	32.6
50–64	25,731	35.3
≥ 65	8054	11.0
**Residency**	**99,367**	
Visitor	87,146	87.7
Saudi resident	2362	2.4
Saudi national	9608	9.7
Unknown	251	0.2
**Hajj status**	**99,367**	
Hajj pilgrim	94,756	95.4
Non-Pilgrim	4611	4.6
**Nationality**^**a**^	**99,367**	
Africa	21,180	21.3
Americas	1567	1.6
Eastern Mediterranean Region	60,394	60.8
Europe	3580	3.6
Other	53	0.1
South-East Asia	10,812	10.9
Western Pacific Region	1781	1.8
**Facility location**	**99,367**	
Arafat (20 PHCCs)	9102	9.2
Mina (25 PHCCs)	84,687	85.2
Muzdalifah (6 PHCCs)	5578	5.6

*PHCC* primary healthcare center

^a^According to the World Health Organization (WHO) regions

### Pattern of diseases

During the study period, 78,105 diagnoses were made, ten of which accounted for over 99% of all diagnoses (Table 2). Diseases of the respiratory system were the most commonly reported (45.0%); of which 72.7% were related to upper respiratory tract infections (URIs). Diseases of the musculoskeletal system and connective tissue accounted for 17.2% of diagnoses (mainly muscle strain (33.6%), joint and lower back pain (29.0%) and, arthritis (16.8%)). Diseases of the skin and subcutaneous tissue represented 10.5% of diagnoses (mainly dermatitis (34.6%)). Few patients (4.0%) had multiple diagnoses. In general, the pattern of disease among pilgrims was similar across locations, although some differences were noted (Table 2). For instance, diseases of the skin and subcutaneous tissue were the second most common diagnoses in Muzdalifah PHCCs, while being ranked third in Mina and Arafat PHCCs.

**Table 2:** The most common diagnosis among patients attending the 51 seasonal primary healthcare centers in the holy sites during the 2019 Hajj

Diagnosis according to ICD-10 classification	%			
	All (***n*** = 78,105)	Mina (***n*** = 65,731)	Arafat (***n*** = 7791)	Muzdalifah (***n*** = 4583)
Diseases of the respiratory system	45.0	46.6	39.2	31.4
Diseases of the musculoskeletal system and connective tissue	17.2	17.5	12.9	20.0
Diseases of the skin and subcutaneous tissue	10.5	9.4	12.7	23.2
Symptoms, signs and abnormal clinical and laboratory findings, not elsewhere classified	8.3	7.7	12.5	8.8
Diseases of the digestive system	7.9	7.8	9.4	7.3
Injury, poisoning and certain other consequences of external causes	4.4	4.4	5.0	2.9
Endocrine, nutritional and metabolic diseases	1.7	1.6	2.6	1.2
Diseases of the eye and adnexa	1.6	1.6	1.8	1.9
Diseases of the circulatory system	1.5	1.5	1.9	1.0
Diseases of the genitourinary system	1.1	1.1	0.8	1.8

*ICD-10* 10th revision of the International Classification of Diseases

### Medications

Patients were prescribed 223,964 medications, mostly at the Mina PHCCs (86.7%) (Table 3). The most prescribed classes of medications were analgesics (25.1%), antibacterials for systemic use (16.5%), anti-inflammatory and antirheumatic products (16.4%), and cough and cold preparations (11.9%). Only 1.3% of the medications were injectable, and 20.8% were antibiotics. Most of the latter were broad-spectrum, in particular amoxicillin (59.6%) and azithromycin (15.8%). In general, the prescribed drugs represented 221 different medications, of which 20 accounted for 91.2% of all medications prescribed during the study period (Table 4). Of these 20 medications, 65.0 and 30.0% were oral and topical medications, respectively, and none were injectable.

**Table 3:** Distribution of patients, encounters and prescribed medications in the 51 seasonal primary healthcare centers in the holy sites during the 2019 Hajj

PHCC (n)	Patients		Encounters		Medications	
	n	%	n	%	n	%
Mina (25)	84,687	85.2	84,754	85.2	194,305	86.7
Arafat (20)	9102	9.2	9110	9.2	18,549	8.3
Muzdalifah (6)	5578	5.6	5580	5.6	11,110	5
**Total**	**99,367**	**100**	**99,444**	**100**	**223,964**	**100**

*PHCC* primary healthcare center

**Table 4:** The 20 most common medications prescribed to patients attending the 51 seasonal primary healthcare centers in the holy sites during the 2019 Hajj

Rank	Medication name	Form	ATC	ATC classification	n	%
1	Paracetamol	Oral	N02	Analgesics	51,257	22.9
2	Amoxicillin Trihydrate	Oral	J01	Antibacterials for systemic use	26,596	11.9
3	Ibuprofen 400 mg Tablet	Oral	M01	Antiinflammatory and antirheumatic products	19,794	8.8
4	Dextromethorphan Hydrobromide	Oral	R05	Cough and cold preparations	17,331	7.7
5	Chlorpheniramine Maleate	Oral	R06	Antihistamines for systemic use	15,177	6.8
6	Diclofenac	Oral/Topical	M01	Antiinflammatory and antirheumatic products	13,785	6.2
7	Azithromycin	Oral	J01	Antibacterials for systemic use	7132	3.2
8	Diphenhydramine, Ammonium Chloride & Sodium Citrate	Oral	R05	Cough and cold preparations	6984	3.1
9	Aluminum-Magnesium Hydroxide	Oral	A02	Drugs for acid related disorders	5893	2.6
10	Naphazoline Hydrochloride & Chlorpheniramine	Nasal	R01	Nasal preparations	5374	2.4
11	Hyoscine Butylbromide	Oral	A03	Drugs for functional gastrointestinal disorders	5114	2.3
12	Hydrocortisone	Topical	D07	Corticosteroids, dermatological preparations	4951	2.2
13	Paraffin	Topical	D02	Emollients and protectives	4490	2.0
14	Magnesium Silicate	Oral	A02	Drugs for acid related disorders	3876	1.7
15	Metronidazole	Oral	P01	Antiprotozoals	3200	1.4
16	Silver Sulfadiazine	Topical	D06	Antibiotics and chemotherapeutics for dermatological use	3068	1.4
17	Salbutamol	Inhalation	P03	Drugs for obstructive airway diseases	3013	1.3
18	Fusidic Acid	Topical	D06	Antibiotics and chemotherapeutics for dermatological use	2742	1.2
19	Calamine	Topical	D02	Emollients and protectives	2575	1.1
20	Rehydration Salt	Oral	A07	Antidiarrheals, intestinal antiinflammatory/antiinfective agents	1910	0.9

*ATC* Anatomical Therapeutic Chemical

### WHO indicators for drug use in PHCCs

**Fig. 2:**
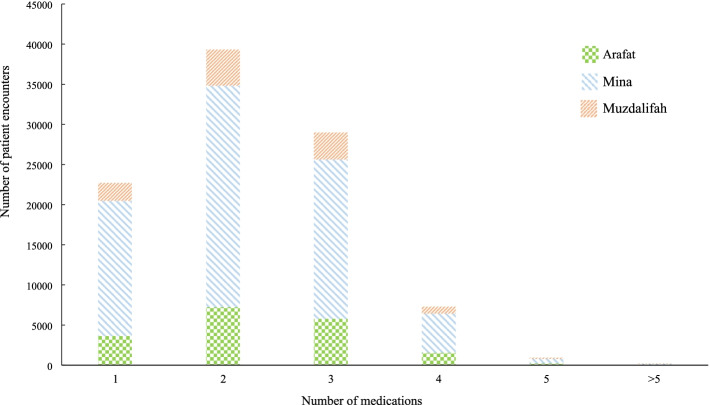
Number of medication prescribed per patient encounter at the 51 seasonal primary healthcare centers in the holy sites during the 2019 Hajj

During the study period, there were 99,444 patient encounters at the 51 PHCCs where medications were prescribed (Table 3). WHO drug use indicators that were possible to calculate from the available data are presented in Table 5. An average of 2.25 (SD = 0.94) medications were prescribed to patients per consultation. This value was lowest in Muzdalifah PHCCs (1.99; SD = 0.88) and highest in Mina PHCCs (2.29; SD = 0.94). In most encounters, 2 or 3 medications were prescribed (Fig. 2). The proportions of encounters with 1, 2, 3, and 4 medications prescribed were 22.8, 39.5, 29.1, and 7.3% respectively. Polypharmacy was rare, observed in 1.3% of the encounters, while excessive polypharmacy was recorded in only one encounter (0.001%).

**Table 5:** WHO core drug use indicators for the 51 seasonal primary healthcare centers in the holy sites during the 2019 Hajj

	PHCCs location (n)			
Indicator	Mina (25)	Arafat (20)	Muzdalifah (6)	All (51)
**Prescribing indicators**				
Average number of drugs per encounter (±SD)	2.29 (0.94)	2.04 (0.93)	1.99 (0.88)	2.25 (0.94)
% of encounters with an antibiotic prescribed	45.8	31.2	30.4	43.6
% of encounters with an injection prescribed	2.52	4.41	2.04	2.67
% of drugs prescribed from essential drugs list or formulary	100	100	100	100
**Patient care indicators**				
% of drugs actually dispensed	92.6	92.0	96.2	92.7
Average time (min) between prescribing and dispensing (±SD)^a^	7.8 (40.8)	10.2 (49.5)	8.36 (36.8)	8.06 (41.4)
**Health facility indicator**				
Availability of copy of essential drugs list or formulary	Yes	Yes	Yes	Yes

*SD* standard deviation, *PHCC* primary healthcare centers

^a^not a WHO drug use indicator

An antibiotic and an injectable were prescribed in 43.6 and 2.67% of patient encounters respectively. Mina PHCCs had the highest percentage of encounters where an antibiotic was prescribed (45.8%), while Arafat PHCCs had the highest percentage of encounters where an injectable was prescribed (4.41%). In all PHCCs, a copy of the essential drugs list was available, and all the prescribed drugs appeared on that list. Most (92.7%) of prescribed drugs were actually dispensed at the PHCCs (Table 5). On average, medications were dispensed 8.06 min (SD = 41.4) from the time they were prescribed for patients. It took slightly longer to dispense medications in Arafat PHCCs (10.2 min; SD = 49.5). Most (91.6%) of the medications were dispensed within 10 min of being prescribed, 79.7% within 5 min, and only 1.5% were dispensed over 1 h from the time they were prescribed.

## Discussion

In the current study, nearly 100,000 pilgrims originating from 186 different countries visited PHCCs in the holy sites during a one-week period; a reflection of the large size and international nature of the Hajj. Yet, pilgrims were mainly older males and from Egypt, Nigeria, Pakistan, KSA, and India. These results are similar to what have been reported previously from PHCCs and hospitals’ OPDs during Hajj [5, 9, 13, 14]. In one study, patients seeking health services during the 2008 Hajj at PHCCs in Mina originated from 82 countries (particularly Egypt (18.9%), Pakistan (17.5%), KSA (8.3%), Nigeria (6.7%) and India (5.7%)), 70.7% were male, and 42.8% were 45–64 years old [9]. Hajj attracts over 2 million pilgrims, a large proportion of which are older males and over half originate from eight countries: Indonesia, Pakistan, India, KSA, Bangladesh, Turkey, Egypt and Nigeria. The demographics of patients attending PHCCs in our study are a reflections of the Hajj population as well as other potential factors, including proximity of PHCCs to pilgrims’ camps, knowledge regarding PHCCs location and opening hours and availability of health services in pilgrims’ camps [15]. The latter may be a key reason for the comparatively small proportion of pilgrims from Indonesia and Turkey attending PHCCs in our study, which has also been reported elsewhere [9, 15].

Similar to previous findings from PHCCs and hospital OPDs during Hajj [5, 9, 10, 14], most patients in our study were seen in Mina facilities and towards the end of the Hajj, post the day of Arafat. As part of the Hajj rituals, pilgrims spent most of the study period in Mina with only a short stay in Arafat and Muzdalifah. In addition, pilgrims are more likely to suffer health issues post the day of Arafat as the physically demanding activities in crowded environments start to take their toll on pilgrims’ health. Alzahrani and colleagues [9] reported that the highest caseload of patients attending Mina PHCCs was 2 days after the day of Arafat. During the latter day, Mina PHCCs received the lowest number of patients, which is understandable given that most pilgrims spend the day in the plane of Arafat; hence most PHCC visits occur at Arafat PHCCs, as also seen in our results.

During the study period, most pilgrims had a single diagnosis, with only 4% having multiple diagnoses. This is comparable to what has been reported from Mina PHCCs during the 2008 Hajj, albeit in that study, the proportion of patients with multiple diagnoses (20.2%) was higher than in our study [9]. However, the pattern of disease was similar to what we reported, with respiratory, musculoskeletal, and skin diseases representing the greatest burden [9]. Respiratory diseases, particularly URIs, were very common regardless of the PHCCs’ location, as evidenced by other studies of primary healthcare services during Hajj [4, 9, 10, 13, 14]. Respiratory tract infections are the leading cause of infectious diseases in Hajj pilgrims, with a prevalence of 50–93% [16]. Respiratory disease accounted for 49 and 61% of diagnoses at Mina PHCCs in 1998 and 2008, respectively [9, 10]. The Hajj environment, which includes crowds, adverse weather and cramped accommodations, can result in increased rates of respiratory diseases and facilitate their spread among pilgrims.

The physically demanding Hajj activities, including walking long distances, carrying heavy weights and uncomfortable sleeping conditions, often in crowded settings and exposed to the outside environment, can contribute in the development of musculoskeletal and skin ailments. As such, these diseases are common among pilgrims [17, 18], including those seeking primary healthcare during the event [4, 9, 10, 13, 14]. In our study, musculoskeletal and skin diseases accounted for respectively 17.2 and 10.5% of diagnoses, which is comparable to the 17.6 and 15.0% reported from Mina PHCCs in the 2008 Hajj [9]. Skin diseases were the second most common diagnoses in Muzdalifah PHCCs, while being ranked third in Mina and Arafat PHCCs. This may be the result of pilgrims spending most of the day of Arafat in the outside environment and exposed to the elements before moving to Muzdalifah. Dermatitis of various etiologies was the most common skin condition (34.6%), which is in accordance with a previous report which found that dermatitis accounted for 23.6% of skin diseases among pilgrims in 1998 [17]. Cardiovascular and metabolic diseases, such as diabetes, represented a small proportion of diagnoses, although these conditions are relatively common among pilgrims [19]. This is in line with other reports from PHCCs during Hajj [9, 10, 14]. A possible reason for this observation is that not all pilgrims with chronic illnesses seek medical care unless their conditions decorate or they run out of medications [20]. Also, health issues linked to these conditions typically require a higher level of medical care [4, 21]. For example, among Indian pilgrims in 2016, cardiovascular disease represented 4.6% of primary care morbidity but 27.1% of tertiary care referrals [4].

The pattern of medications prescribed to patients was consistent with the pattern of diseases and caseload at various PHCCs. The majority of medications were prescribed at Mina PHCCs, with the common classes being analgesics (25.1%), antibacterials for systemic use (16.5%), anti-inflammatory and antirheumatic products (16.4%), and cough and cold preparations (11.9%). This is in line with data from both PHCCs and hospitals OPDs during the pilgrimage as well as from Hajj medical mission camps [4, 5, 9, 13]. For example, the most frequently prescribed drugs to patients attending 13 PHCCs in Mina in 2008 Hajj were analgesics and antipyretics (79.4%), followed by antibiotics (53.9%) and cough syrups (37.1%) [9]. In another report, anti-inflammatory and antirheumatic products (22.9%), analgesics (22.8%) and antibacterials for systemic use (17%) were the most commonly prescribed medications for hospital outpatients during the 2018 Hajj [5]. Similarly, data from a Makkah hospital OPD during the 2003 Hajj found that the most common medications dispensed were antibiotics (43.3%), followed by analgesics and antipyretics (25.2%) [13].

We assessed the drug utilization performance of PHCCs using a number of WHO indicators that covered prescribing, patient care, and health facilities parameters [11]. For the prescribing indicators, the average number of medications per encounter was 2.25 (SD = 0.94). This is higher than the proposed admissible range of 1.6–1.8 [22], but in line with previous reports from Mina PHCCs (2.35; SD = 0.97), Hajj seasonal hospitals OPDs (2.6; SD = 1.2) and non-Hajj PHCCs in KSA (2.4; SD = 1.2) [5, 9, 23]. Most patients (77.2%) received multiple therapies, although polypharmacy (≥5 medications) was observed in a very small proportion of encounters (1.3%). Similar to our findings, most patients attending Mina PHCCs during the 2008 Hajj (80.2%) and outpatients visiting Hajj hospitals in 2018 (84.4%), received multiple medications [5, 9]. In the latter study, polypharmacy was reported in 4.8% of encounters [5]. In general, prescribing of multiple medications is common in an older population with multiple morbidities. Given the unique nature of Hajj and the high prevalence of older pilgrims, many with underlying health conditions [19], it is expected that many pilgrims would be on multiple therapies. Yet, the low rate of polypharmacy is reassuring given that the latter is associated with negative outcomes including adverse drug reactions, failure to comply with treatment, mortality, and financial loss [24–26].

In the current study, the percentage of patients’ encounters with an antibiotic prescribed was 43.6%, which is higher than the proposed optimal range (20–26.8%) [22, 27]. However, the value is consistent with the frequent prescribing and use of antibiotics during the Hajj. Data from PHCCs, hospital OPDs and medical mission camps report that 43.3–53.9% of pilgrims were prescribed antibiotics [4, 5, 9, 13]. Antibiotic use in Hajj is common, with up to 58.5% of pilgrims having used these medications during the event [28]. Many antibiotics are prescribed empirically during Hajj, especially in primary-care settings, with a tendency to prescribe broad-spectrum agents given a limited time to assess patients and to cover suspected infections. High prevalence of antibiotics use is however not unique to Hajj. One study found that in over half of the 44 countries investigated, more than 50% of patients were treated with antibiotics [29]. The latter study also reported that the percentage of patients treated with an injection ranged between 10 and 57% in most countries [29]. In our study, this percentage was 2.7%, which is lower than reported from outpatients clinics in Hajj hospitals (6.5%) [5], as well as the proposed optimal range for this indicator (13.4–24.1%) [22, 27]. Beyond being uncomfortable for patients and more costly, excessive use of injections can result in a higher risk of bloodborne diseases [30].

For the final prescribing indicator determined in this study, we found that all of the prescribed drugs appeared on the essential drug list available in the PHCCs. This is at the higher end of the range reported from other countries (58–100%), and similar to values from Hajj seasonal hospitals (100%) and non-Hajj PHCCs in KSA (99.2%) [5, 23, 29]. These results are not surprising given that during Hajj, physicians only prescribed medications from the list available in the PHCCs’ pharmacy electronic system. As part of the Hajj health services readiness, all Hajj seasonal hospitals and PHCCs have a list of all medications available and stocked at each facility before they become operational. As such, all PHCCs also had a copy of essential drug list/formulary, which is the optimal value (100%) for this facility-specific indicator [27, 31]. This is similar to what was reported from seasonal Hajj hospitals [5], but slightly higher than findings from non-Hajj PHCCs in other parts of the Kingdom (90%) [32].

For patient-care indicators, we found that the percentage of medicines actually dispensed in our study was 92.7%. This is comparable to what was reported from seasonal Hajj hospitals (90.0%), non-Hajj PHCCs in KSA (99.6%), and close to the optimal value of 100% [5, 27, 32]. In general, unavailability of medication is the main reason for lower values of this indicator [33]. However, given the way medications are provided to, and managed within, seasonal health facilities during Hajj, it is likely that other factors may explain the non-optimal value of this indicator in our study. These include medication not dispensed for patients who have already been dispensed the same medications in previous visits to the same PHCCs or other healthcare facilities in the holy sites, medications dispensed but not entered into the PHCCs’ pharmacy electronic system, or pilgrims simply not picking up their prescribed medications [5]. Given the retrospective design of the current study and the available data, we were unable to calculate the WHO indicator; average dispensing time for medications. However, we calculate a proxy indicator which is the average time between prescribing and dispensing of medications in the PHCCs. This value was 8 min (SD = 41.4), which is half of that reported from hospitals in the same locations (16.4 min; SD = 119.8) [5]. However, similar to the latter study, we found that most medications were dispensed within 10 min of being prescribed, with health facilities in Arafat being the slowest. This can be explained by the fact that these facilities have a much higher rate of encounters per working hours, given that they serve the entirety of the Hajj population for a single day [5].

The present study has some limitations. First, the study was conducted in 51 seasonal PHCCs in the holy sites and was limited to mainly the Hajj ritual days. Pilgrims attend 139 PHCCs in Makkah and in Medina throughout the 2-month Hajj season [2]. Therefore, our results may not reflect the pattern of disease and medication prescribing in all PHCCs throughout the Hajj season. However, our study included 55% of PHCCs in the holy sites [2], and our results are similar to those reported from studies of randomly selected holy sites PHCCs [9, 10]. Therefore, our findings are an appropriate reflection of the situation in Hajj seasonal PHCCs. Second, pilgrims may acquire medication through various health facilities and private pharmacies available in Hajj, including their countries’ medical missions, or use medication brought from home countries. Hence, our results reflect only part of the overall medication use and pattern among pilgrims during Hajj. Finally, based on the available data, only some of the WHO drug use indicators could be calculated.

In summary, we reported on the pattern of disease, caseload, and medication prescribing and dispensing at seasonal PHCCs during the 2019 Hajj mass gathering. Most patients were seen in Mina and post the day of Arafat. Respiratory diseases, particularly URIs, were the most common diagnoses, followed by musculoskeletal and skin diseases. The most prescribed classes of medications were analgesics, antibiotics, anti-inflammatory and antirheumatic products, and cough and cold preparations. Our results provide policymakers and Hajj stakeholders with the evidence base to optimize planning, resources allocation and delivery of healthcare services during Hajj. Some recommendations can be made. For example, healthcare resources, including staff and consumables, should be mobilized to diagnose and treat respiratory, musculoskeletal and skin diseases, and to prepare Mina PHCCs for the influx of patients post the day of Arafat. Ensuring accessibility to healthcare at pilgrims’ camps, through the pilgrims’ medical missions or dedicated healthcare officers, would reduce the load on PHCCs in the holy sites. Similarly, given the large burden of respiratory infections and the common use of antibiotics, introduction of point-of-care molecular diagnostics would ensure rapid diagnosis of these infections and identification of their causative agents, reducing unnecessary antibiotic prescribing. Our study is the first report of some of the WHO indicators for drug use in Hajj PHCCs. Given the unique context of Hajj and its population, objective norms for the indicators calculated in this study do not exist, and our values are not comparable to optimal values reported for non-mass gatherings settings [22, 27, 31]. Therefore, indicators reported in this study, along with findings from seasonal hospitals in Hajj [5], can be used to guide standards for medication prescribing and use during Hajj and other mass gatherings, and develop optimal values for these indicators in such contexts.

## Data Availability

The datasets used and analysed during the current study are available from the corresponding author on reasonable request.
